# Case Report: MR-Guided Adaptive Radiotherapy, Some Room to Maneuver

**DOI:** 10.3389/fonc.2022.877452

**Published:** 2022-04-14

**Authors:** Winnie Li, Jeff Winter, Jerusha Padayachee, Jennifer Dang, Vickie Kong, Peter Chung

**Affiliations:** ^1^ Radiation Medicine Program, Princess Margaret Cancer Centre, Toronto, ON, Canada; ^2^ Department of Radiation Oncology, University of Toronto, Toronto, ON, Canada

**Keywords:** MR-Linac, adaptive radiotherapy, prostate cancer, ultrahypofractionation, inter-fraction motion

## Abstract

**Background:**

A magnetic resonance linear accelerator (MR-Linac) provides superior soft tissue contrast to evaluate inter- and intra-fraction motion and facilitate online adaptive radiation therapy (ART). We present here an unusual case of locally advanced castrate-resistant prostate cancer treated with high-dose palliative ultra-hypofractionated radiation therapy on the MR-Linac with significant inter-fraction tumor regression.

**Case Presentation:**

The patient was a 65-year-old man diagnosed with metastatic prostate cancer to bone and pelvic lymph nodes 7 years prior. At diagnosis, he presented with a PSA of 23 ng/ml and was commenced on a luteinizing hormone-releasing hormone agonist, achieving a PSA nadir of 4.68 ng/ml at 12 months. The patient subsequently had progressive lower urinary tract symptoms, his PSA increased to 47 ng/ml, and there was a markedly enlarged pelvic mass involving the prostate with gross extra-capsular disease and invasion into the posterior bladder wall. The patient was referred for palliative radiation to the pelvic mass due to urinary symptoms, pain, and lower limb paraesthesia. Treatment was planned to be delivered on the MR-Linac with a schedule of 36 Gy over 6 weekly factions allowing for maximal target dose delivery while minimizing surrounding organs at risk (OARs) radiation exposure. Unexpectedly, the target volume had a marked 49% (453 cc to 233 cc) reduction that was accounted for in the online adaptive process. A new reference plan was generated after 3 fractions to add sacral plexus as an OAR, previously not visible due to mass encroachment. The patient reported ongoing reduction in urinary symptoms, pelvic pain, and lower limb paresthesia by the end of treatment.

**Conclusion:**

Using daily MR-guided ART, improved visualization of the changing target and OARs ensured safe dose escalation. The unexpected positive response of the target and improved patient outcomes demonstrated the added value of the MR-Linac for online adaptive radiotherapy in this setting.

## Introduction

Image-guided radiation therapy (IGRT) enables the visualization, quantification, and correction of patient setup errors, monitoring changes to ensure high-quality dose delivery (1, 2). Recent advances in radiation therapy have introduced the clinical availability of a hybrid magnetic resonance linear accelerator (MR-Linac) to evaluate inter- and intra-fraction motion (3–5). Providing superior soft tissue contrast, these systems enable online adaptive radiation therapy (ART) to account for spatial and temporal anatomic changes to maximize dose to target while minimizing dose to surrounding organs at risk (OARs) (6, 7).

As prostate cancer is characterized by a low α/β ratio, hypofractionation or stereotactic body radiation therapy is increasingly prevalent to improve radiation efficacy while minimizing toxicity (8–10). The clinical implementation of hypofractionated prostate MR-Linac ART has been widely reported in the literature with promising early results (11–14). Though associated with a longer treatment session, use of ART with the MR-Linac enables tailored dose delivery to the target through re-contouring and re-planning activities prior to each fraction.

We present here an unusual case of a patient with metastatic castrate-resistant prostate cancer requiring palliative pelvic radiotherapy that resulted in large volume inter-fraction tumor regression that demonstrated a role of the MR-Linac in online adaptation.

## Case Description

The patient was a 65-year-old man diagnosed with metastatic prostate cancer to bone and pelvic lymph nodes 7 years prior. At the time of diagnosis, he presented with a prostate-specific antigen (PSA) of 23 ng/ml, and prostate biopsies confirming Gleason grade 4 + 5 = 9 disease with 12/12 cores involved and 80% overall involvement. Staging bone scan and CT thorax, abdomen and pelvis demonstrated multiple bone metastases and pelvic lymphadenopathy. The prostate was described as enlarged and heterogeneous with irregular margins. He commenced on a luteinizing hormone-releasing hormone (LHRH) agonist and achieved a PSA nadir of 4.68 ng/ml 1 year following androgen deprivation therapy (ADT) institution.

**Figure 1:**
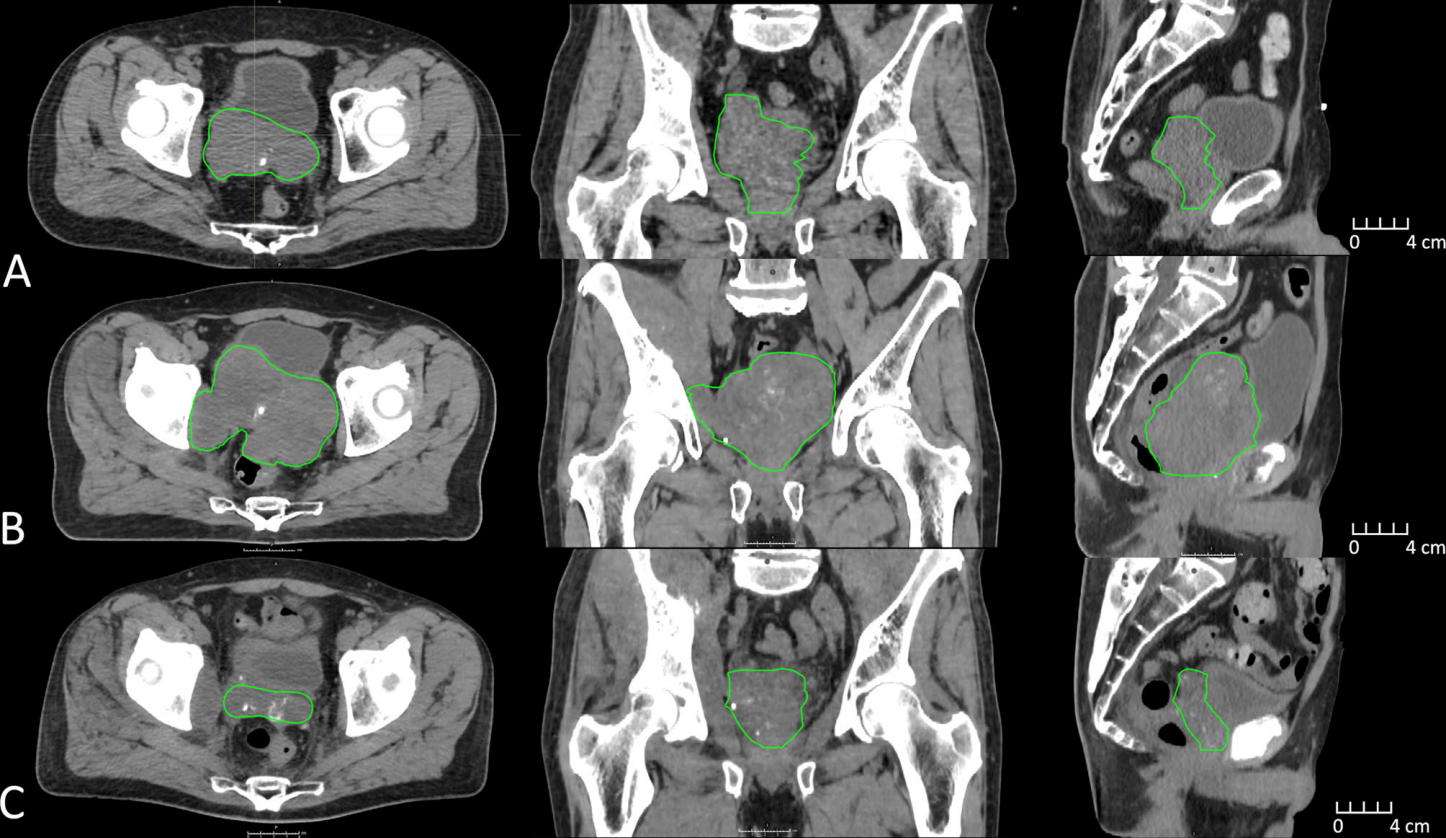
CT imaging depicting clinical target volume (green) changes approximately 1 year prior to MR-Linac treatment **(A)**, immediately prior to MR-Linac treatment **(B)**, and 6 months post MR-Linac treatment **(C)**.

The patient subsequently had progressive lower urinary tract symptoms with nocturia x 5 and weak stream. His PSA had risen to 47 ng/ml and re-staging CT showed progressive bone metastases together with soft tissue disease and a 5.5-cm left adrenal metastasis. Additionally, there was a markedly enlarged pelvic mass centered on the prostate with gross extra-capsular extension and invasion into the posterior bladder wall (**Figure 1A**). Enzalutamide was commenced, with an initial biochemical and radiological response. However, this was transient and within 4 months of commencing enzalutamide, PSA started rising again and peaked at 60 ng/ml associated with imaging progression. The imaging revealed multiple lobulated masses arising from the prostate encroaching onto the rectum, and new intramuscular masses in the right iliopsoas and right obturator internus (**Figure 1B**). Coinciding with this, the patient reported symptoms of pelvic pain and lower limb paresthesia. Enzalutamide was stopped and he was referred for palliative radiotherapy to the prostate. After discussion, it was agreed to proceed with radiotherapy, but as the mass was so large, it was felt that an ultra-hypofractionated approach might be beneficial. MR-Linac treatment was thought to offer the most ability to tailor radiotherapy delivery to maximize dose delivery but minimize dose to OARs.

The patient underwent both CT and MRI simulation session for treatment planning. A high-resolution T2-weighted MR image acquired on the Unity MR-Linac (Elekta Unity, Stockholm, Sweden) was used for reference planning, and the CT image was used to provide electron density information. The target (clinical target volume, CTV), bladder, rectum, and large bowel were contoured by the radiation oncologist (RO), and a planning target volume (PTV) was created using a 5-mm uniform expansion around the CTV. A reference plan of 36 Gy over 6 weekly factions was generated in the MR-Linac treatment planning system (Monaco v5.4, Elekta AB, Stockholm, Sweden) using a 9-field IMRT technique. The plan derived was purposefully heterogeneous allowing central tumor region dose escalation to 48 Gy. The OARs assessed included rectum, large bowel, and small bowel where dose constraints were strictly observed. The majority of the dose-escalated tumor mass was posterior–superior and no attempt was made to include the urethra in the dose-escalated volume. It was observed at fraction 2 that there had been substantial target volume reduction (17%) mostly in the superior and posterior directions. A new reference plan was generated after 3 fractions to add the sacral plexus contours and constraints (previously not visible due to mass encroachment) and also accounted for the changing tumor mass.

For each MR-Linac treatment session, a localization T2-weighted MR image (MR_Loc_) was acquired. The reference plan was used as a starting point to generate an adapted plan based on the contours redefined in-session on the MR_Loc_ by the RO. While quality control checks, including secondary monitor unit verification and multi-disciplinary plan quality review on the adapted plan were performed, a verification MR (MR_Ver_) was acquired to ensure the target was encompassed within the PTV. A third beam on MR (MR_BO_) was acquired during radiotherapy beam on, which allowed assessment of the internal anatomy during treatment delivery. The patient completed the patient-reported outcome tool, Expanded Prostate Cancer Index Composite for Clinical Practice (EPIC-CP), prior to each treatment.

To determine the delivered dose for each treated fraction, the clinically treated daily adapted beams were computed on the MR_BO_. To simulate the delivered dose without daily adaptation, we computed reference plan beams on the MR_BO_. The delivered dose without daily adaptation using the initial reference plan for the first three fractions and the updated reference plan for the final three fractions were simulated. All images, structures, and dose distributions were exported to a separate treatment planning system for dose accumulation (Raystation v8, RaySearch, Stockholm, Sweden) using deformable image registration (DIR). To generate a high-quality DIR, manual contours were generated on the MR_BO_ images and these contours were used as controlling regions of interest (ROIs) for the hybrid intensity and structure-based DIR between the reference MR and each MR_BO_. Following manual review of the DIR quality, dose for each adapted fraction as well as the simulated no-adaptation dose was deformed to the reference MR using the deformed vector field for evaluation on the reference MR.

**Figure 2:**
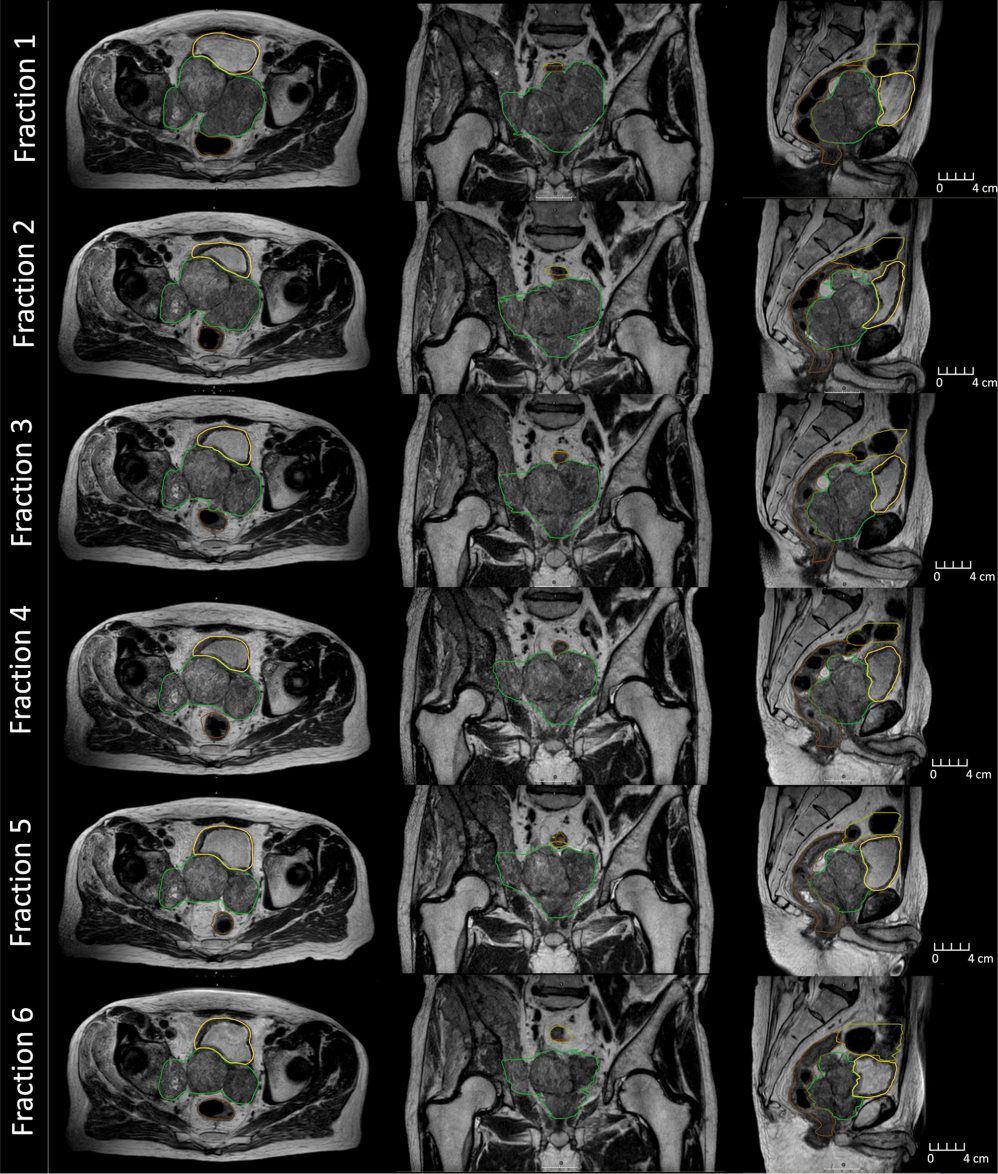
MR images collected during beam delivery for each fraction on the MR-Linac during treatment. Displayed is the clinical target volume (green), rectum (brown), bladder (yellow), and large bowel (olive).

**Figure 3:**
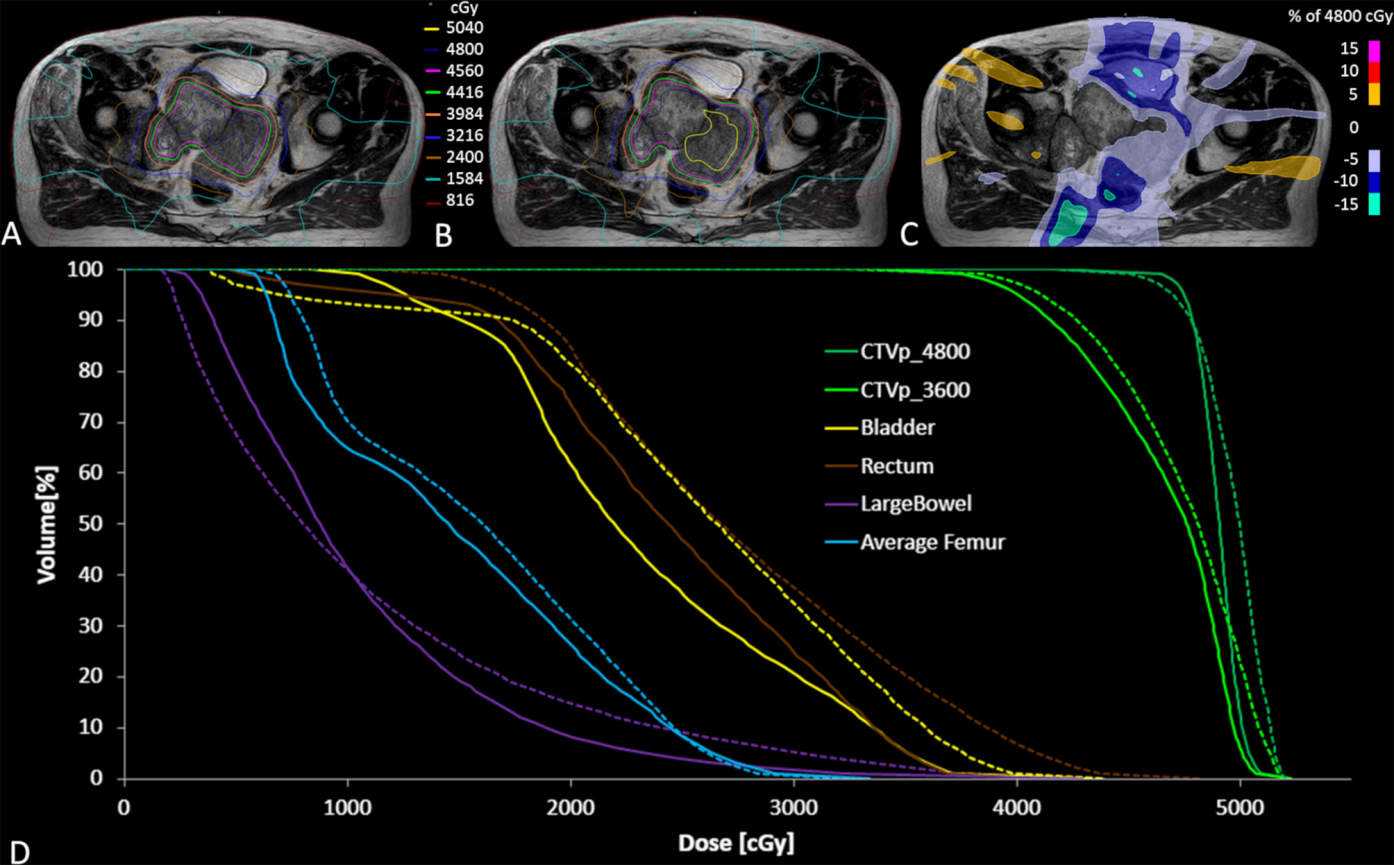
Accumulated dose for all fractions on the reference MR scan for the clinically delivered treatment plan **(A)**, and simulated dose distribution without daily adaptation **(B)** along with the dose difference for the adapted plan minus non-adapted plan **(C)**. The accumulated DVH curves for both the adapted (solid lines) and non-adapted (dotted lines) are also illustrated **(D)**.

The average MR-Linac treatment time was 64 min (range 59–77 min). Over the course of 6 fractions, the CTV decreased in volume from 453 to 233 cc (49%) (**Figure 2**). Using dose accumulation, the demonstrated cumulative dose to OARs was reduced with the use of daily adaptation compared with the simulated single offline adaptation (**Figure 3**). Both daily adaptation and offline adaptation provided sufficient target coverage, but with offline adaptation, target doses were greater than intended and exceeded the maximum dose to 1 cc clinical goal. Use of daily adaptation resulted in lower OAR doses as we were able to progressively spare the OARs as the target mass decreased (**Table 1**). In particular, dose reduction to the bladder, rectum, and large bowel was substantially reduced with use of online adaptation. There was a 16% reduction in D5cc bladder and 12% reduction in D20 rectum in plans with adaptation versus without adaptation.

**Table 1:** Comparison of key dose volume histogram (DVH) metrics between daily adapted and simulated workflow with single mid-treatment adaptation.

Region of Interest	DVH Metric	Clinical Goal (cGy)	Daily Adapted (cGy)	Simulated No Daily Adaptation (cGy)
CTVp_4800	D95	4,560	4,576	4,698
CTVp_3600	D99	3,420	3,570	3,849
CTVp_3600	D1cc	5,040	4,869	5,193
Rectum	D50	1,350	2,193	2,654
Rectum	D20	2,190	2,928	3,334
Rectum	D1cc	3,600	3,422	3,903
Bladder	D40	1,350	2,343	2,922
Bladder	D5cc	3,600	3,480	4,139
Left Femur	D5	1,520	1,967	2,058
Right Femur	D5	1,520	2,251	2,160
Large Bowel	D1cc	2,530	3,166	3,835
Left Sacral Plexus*	D1cc	1,800	1,510	1,792
Right Sacral Plexus*	D1cc	1,800	1,402	1,475

*Accumulated dose for the final 3 fractions reported, as the relevant region of the sacral plexus was only visible after fraction 3. Also note that the clinical goals were based on three fractions only.

Treatment was well tolerated with no patient-reported acute toxicities. Patient-reported outcomes collected through EPIC-CP indicated no worsening urinary and bowel symptoms throughout treatment. More so, urinary symptoms improved during treatment, corresponding with treatment response. The score for need to urinate frequently became a small problem by fraction 2, reduced to a very small problem by fraction 4, and resolved to no problems for fractions 5 and 6. The patient experienced very small problems with hot flashes and feeling depressed for the first 3 fractions, resolved to no problems for either factor over the last 3 fractions. Finally, the patient experienced a very small problem with a lack of energy for the first 4 fractions, increasing to a small problem on fraction 5, and moderate problem on fraction 6.

One month following radiation treatment, the patient’s PSA reduced to 21 and there was evidence of partial radiological response within the prostate (**Figure 1C**). There was, however, progression within the intramuscular masses and left adrenal gland, which were not intentionally a part of the treatment target volume. Biopsies were obtained of the adrenal mass, which confirmed metastatic prostate adenocarcinoma. The patient was referred for consideration of systemic therapy clinical trials and underwent genetic testing as part of the assessment. He was found to carry a BRCA2 mutation, and was subsequently enrolled onto a randomized trial involving a PARP inhibitor. At the last follow-up, there was biochemical and radiological stability of disease.

## Discussion

Compared to conventional linac treatment, MR-guided daily ART provides improved target and OAR visualization with the ability to adapt radiation delivery to account for inter-fraction changes, enabling safe dose escalation. At our institution, one fractionation schedule for metastatic prostate cancer, where the goal of treatment is local pelvic tumor control, is based on one of two dose schedules used in the STAMPEDE trial 36 Gy in 6 weekly treatments (15). With MR-guided daily ART, the central target region in this patient was safely escalated to 48 Gy in an attempt to provide a higher probability of local control in the large pelvic mass without increasing risk of toxicity. With the superior soft tissue contrast at each fraction, it was possible to visualize boundaries between CTV and OARs more clearly, as treatment progressed. This facilitated optimizing the therapeutic ratio, by maximizing target dose while minimizing OAR doses. On a conventional cone-beam CT (CBCT)-guided Linac, weekly changes in the target and OARs for this patient may not have been as readily observed.

Small target volume changes associated with prostate hypofractionation have previously been described with MR-Linac extreme hypofractionation (16, 17). In contrast, our patient had almost a 50% reduction in target volume, potentially due to the longer span in overall treatment time. The reduction may have also been associated with his BRCA2 mutation status as there are reports of increased radiosensitivity in normal and tumor cells of BRCA mutation carriers (18).

Determining the cost-effectiveness of novel radiation therapy technologies is important to balance cost versus perceived clinical benefits (19). The health economics for prostate MR-Linac treatments have been explored, where hypofractionated schedules did not show cost-effectiveness due to its high cost and lack of evidence to show substantial reduction in complications (20). As the current workflow for the MR-Linac involve a multidisciplinary team over a longer period of time when compared to conventional Linac treatment, one of the main implementation challenges is increased human-resource requirements (10, 21–23). Initiatives such as an oncologist-lite or therapist-led workflow to reduce human resource costs are being developed, but have yet to become mainstream practice (24–26). However, for this patient case, there were economic benefits and resource savings by treating him on the MR-Linac. Had the patient been treated on a conventional Linac with CBCT imaging, he may have triggered at least 1 iteration of replanning activity with the target size changes, even with the limited pelvic soft tissue contrast on CBCT. This replanning activity would have been resource intensive, involving the coordination of a new reference scanning session on the CT, recontouring of targets, creation of a new plan, additional physics and quality control checks, and potential delayed timelines for the patient.

There are several practical learnings in this case study to inform future use cases on the MR-Linac. First, temporal and spatial changes with the target and OARs may be difficult to predict in patients with large volume targets. With daily ART, it is possible to safely escalate dose beyond standard dose fractionation schemes to maximize impact on the target volume while minimizing OAR dose and associated toxicities. In this case, the central volume was escalated, but in the future, MR imaging biomarkers such as diffusion-weighted imaging estimates of the apparent diffusion coefficient and intra-voxel incoherent motion collected at each fraction offer potential for biological-based adaptive dose escalation. Second, this case demonstrates the value of the MR-Linac for palliative radiotherapy if target changes are expected, or unpredictable, over a course of treatment. The feasibility of palliative MR-Linac treatments have been explored to improve treatment efficiencies and reduce wait times (27). The unexpectedly large target response and positive cancer and toxicity outcomes supersedes the challenges related to longer treatment sessions and increased resource allocation associated with the MR-Linac. Finally, while the use of MRL appeared to be beneficial in this patient, it remains a relatively expensive, time-consuming treatment method. We would not routinely recommend this for all patients undergoing RT to the primary in the oligometastatic setting or in the setting or “standard” palliative radiotherapy. However, selective use in the situation where there are anticipated benefits in OAR sparing (particularly for ultrahypofractionated treatments) where challenging patient anatomy exists or, as experienced in our patient, large volume changes likely to benefit from adaptive RT may be the most appropriate indications.

## Patient Perspective

Prior to treatment, the patient reported pelvic pains and numbness in his toes. Following fraction 1, there was improvement noted in his presenting symptoms of pain and paresthesia, which completely abated by the end of the treatment course. Over the course of treatment, the patient was able to tolerate longer commutes without experiencing pain. Of note, the patient noted that mid-treatment, he was able to drive a long distance without the need for a break to enjoy a picnic with his wife.

## Data Availability Statement

The original contributions presented in the study are included in the article/supplementary material. Further inquiries can be directed to the corresponding author.

## Ethics Statement

The studies involving human participants were reviewed and approved by the University Health Network Research Ethics Board. The patients/participants provided their written informed consent to participate in this study. Written informed consent was obtained from the individual(s) for the publication of any potentially identifiable images or data included in this article.

## Author Contributions

WL wrote the first draft of the manuscript. JW, JP, and PC wrote sections of the manuscript. WL, JD, and VK contributed to image review and contouring. JW performed dose accumulation and generated figures. All authors contributed to manuscript revision, read, and approved the submitted version.

## Conflict of Interest

The authors declare that the research was conducted in the absence of any commercial or financial relationships that could be construed as a potential conflict of interest.

## Publisher’s Note

All claims expressed in this article are solely those of the authors and do not necessarily represent those of their affiliated organizations, or those of the publisher, the editors and the reviewers. Any product that may be evaluated in this article, or claim that may be made by its manufacturer, is not guaranteed or endorsed by the publisher.
